# Effect of Lockdown and Unlock Following COVID-19 on the Presentation of Patients With Endophthalmitis at a Tertiary Eye Center Over One Year

**DOI:** 10.7759/cureus.19469

**Published:** 2021-11-11

**Authors:** Anthony Vipin Das, Vivek Pravin Dave

**Affiliations:** 1 Department of eyeSmart Electronic Medical Record (EMR) & AEye, Indian Health Outcomes, Public Health and Economics Research Center, L V Prasad Eye Institute, Hyderabad, IND; 2 Smt. Kanuri Santhamma Center for Vitreoretinal Diseases, The Retina Institute, LV Prasad Eye Institute, Hyderabad, IND

**Keywords:** unlock, endophthalmitis, big data, india, covid-19 pandemic

## Abstract

Introduction

Endophthalmitis is an emergency where patients need immediate intervention to salvage both vision and the globe. Regular follow-up care and close monitoring of the status of the patient are crucial in determining the final outcomes of the treatment. While there is ample literature on the epidemiology and demography of endophthalmitis across the globe, no information exists on the effect of the coronavirus disease 2019 (COVID-19) pandemic on the presentation patterns of endophthalmitis.

Objectives

To describe the impact of the lockdown and unlock phases of the COVID-19 pandemic on the presentation of endophthalmitis at a tertiary eye care center in India.

Methods

This cross-sectional hospital-based study included 1,253 patients presenting between March 25, 2017, and March 31, 2021. The data on the patients diagnosed with endophthalmitis in one or both eyes presenting during the lockdown and unlock phases were compared with the respective time periods in the previous three years before COVID-19.

Results

The patient numbers dropped to 42.18% (27/64) of pre-COVID-19 volumes during the lockdown phase. There was a near-complete recovery of the outpatient numbers to 85.71% (26/30) of pre-COVID-19 volumes by March 2021. This gradual incremental trend was seen in etiology due to post-surgery (88%) and endogenous (159%) infections while post-traumatic (67%) and post-infection (0%) infections were lower than pre-COVID-19 volumes by March 2021. There was a slight increase in the proportion of patients with acute endophthalmitis (88.93% vs 86.20%; *p=0.76*) presenting during the COVID-19 phase as compared to the pre-COVID-19 phase. The proportion of patients requiring inter-state travel showed complete recovery to 107% of pre-COVID-19 distribution by March 2021.

Conclusion

The first year of the COVID-19 pandemic saw a decline of over half the number of patients with endophthalmitis accessing eye care services. There was an increase in the presentation of endogenous endophthalmitis and a decrease in post-traumatic forms. Patients requiring inter-state travel recovered during the unlock phase.

## Introduction

India recorded one of the most devastating second waves around the world during the coronavirus disease 2019 (COVID-19) pandemic and is now witnessing a gradual decline in infections [1]. The enforcement of a national lockdown in India last year brought new challenges to the fore in access to healthcare services for patients across the country [2]. Studies from India have shown a drastic reduction in outpatient numbers during the early phase of the lockdown [3]. This had possible ramifications on the presentation of patients with endophthalmitis accessing eye care services.

Endophthalmitis is one of the most dreaded eye conditions. Most cases occur either following surgery or post-trauma. It is defined as inflammation of the inner coat of the eyeball primarily involving the vitreous cavity. The organisms gain entry either from exogenous sources like trauma or surgery or from endogenous hematogenous spread from distant sites [4]. Bacterial endophthalmitis is more common than fungal endophthalmitis. Staphylococcus epidermidis is the most common isolate in the USA, Europe, and India [5-6].

Endophthalmitis being an emergency, patients affected with it need immediate intervention to salvage both vision and the globe. Regular follow-up care and close monitoring of the status of the patient are crucial in determining the final outcomes of the treatment. While there is ample literature on the epidemiology and demography of endophthalmitis across the globe, there is no existent information on the effect of the COVID-19 pandemic on the presentation patterns of endophthalmitis. In the current communication, we describe a comparative report of the effect of the lockdown and unlock of the COVID-19 pandemic on the presentation of patients with endophthalmitis at a tertiary eye care center in India.

## Materials and methods

Study design, period, location, and approval

This was a cross-sectional, observational, hospital-based study that included patients presenting between March 25, 2017, and March 31, 2021, to a tertiary eye care center located in India. A standard consent form for electronic data sharing for research purposes was signed by the patient or the parents or guardians of the patient at the time of registration. None of the identifiable parameters of the patient information were used for the analysis of the data. The study adhered to the Declaration of Helsinki and was approved by the Institutional Ethics Committee (LV Prasad Eye Institute Institutional Review Board) with the approval number LEC BHR-R-05-21-659. The clinical data of each patient who underwent a comprehensive ophthalmic examination were entered into a browser-based electronic medical records system (eyeSmart Electronic Medical Record (EMR)) using a standardized template by trained ophthalmic personnel and supervised by an ophthalmologist [7-8].

Data retrieval and processing

A total of 1,253 patients of all ages diagnosed with endophthalmitis in one or both eyes at the tertiary eye care center during the study period were included. A sub-group analysis of the patients based on the etiology of the endophthalmitis (post-surgical, post-traumatic, endogenous, and post-infection) was performed to assess the trends. The data of these patients were retrieved from the electronic medical record database and segregated in a single excel sheet (Microsoft Excel®; Microsoft Corporation, Redmond, WA). Data on patient demographics, ocular diagnosis, and surgical intervention were used for analysis. The Excel sheet with the required data was then used for analysis using the appropriate statistical software. The study duration was divided into three categories: Pre-COVID-19 between March 25, 2017, and March 24, 2020, Lockdown (phases 1-4) between March 25, 2021, and May 31, 2021, and Unlock (phases 1-10) between June 1, 2020, to March 31, 2021 [9]. Geographic categorization was performed in relation to the location of origin of the patient to the eye care center at presentation. The patients presenting from the same location of the eye center were classified as “intra-city,” those from outside the city but from the same state of the eye center were classified as “intra-state,” those from outside the state were classified as “inter-state,” and those from outside India were classified as “international” patients. The demographic distribution and clinical presentation of the patients in these three categories were used for comparative analysis.

Statistical analysis

Descriptive statistics using mean ± standard deviation and median with interquartile range (IQR) were used to elucidate the demographic and clinical data using Microsoft Excel 2019. P-value calculation and subgroup analysis were performed.

## Results

Overall trend of endophthalmitis

Overall, 1,253 patients diagnosed with endophthalmitis in one or both eyes presented during the study period (Table 1).

**Table 1:** Demographics with break-up across the three phases of the endophthalmitis footfall

Variable		n	%	Pre-COVID-19	%	Lockdown (phase 1-4)	%	Unlock (phase 1-10)	%	p value
Total Cases		1253	100.00%	1000	79.81%	27	2.15%	226	18.04%	
Gender	Male	792	63.21%	613	61.30%	18	66.67%	161	71.24%	0.79
	Female	461	36.79%	387	38.70%	9	33.33%	65	28.76%	0.70
Age (years)	0-30	376	30.01%	303	30.30%	9	33.33%	64	28.32%	0.81
	31-40	119	9.50%	88	8.80%	4	14.81%	27	11.95%	0.34
	41-50	145	11.57%	107	10.70%	4	14.81%	34	15.04%	0.55
	51-60	241	19.23%	197	19.70%	7	25.93%	37	16.37%	0.52
	61-70	251	20.03%	206	20.60%	3	11.11%	42	18.58%	0.31
	71-100	121	9.66%	99	9.90%	0	0.00%	22	9.73%	NA
Socio-economic status	Paying	598	47.73%	458	45.80%	12	44.44%	128	56.64%	0.93
	Non Paying	655	52.27%	542	54.20%	15	55.56%	98	43.36%	0.94
Geographic Status	Urban	195	15.56%	155	15.50%	3	11.11%	37	16.37%	0.59
	Rural	655	52.27%	511	51.10%	19	70.37%	125	55.31%	0.29
	Metropolitan	403	32.16%	334	33.40%	5	18.52%	64	28.32%	0.22
Distance to eye care center	Intracity	243	19.39%	191	19.10%	6	22.22%	46	20.35%	0.74
	Intrastate	467	37.27%	377	37.70%	12	44.44%	78	34.51%	0.64
	Interstate	527	42.06%	417	41.70%	9	33.33%	101	44.69%	0.56
	International	16	1.28%	15	1.50%	0	0.00%	1	0.44%	NA
Type of Visit	Fresh	930	74.22%	745	74.50%	18	66.67%	167	73.89%	0.72
	Follow-up	323	25.78%	255	25.50%	9	33.33%	59	26.11%	0.49
Etiology	Post Surgical	555	44.29%	470	47.00%	4	14.81%	81	35.84%	0.024
	Post Traumatic	371	29.61%	288	28.80%	13	48.15%	70	30.97%	0.13
	Endogenous	271	21.63%	193	19.30%	9	33.33%	69	30.53%	0.16
	Post Infection	56	4.47%	49	4.90%	1	3.70%	6	2.65%	0.69
Type of Endophthalmitis	Acute Endophthalmitis	1087	86.75%	862	86.20%	23	85.19%	202	89.38%	0.97
	Chronic Endophthalmitis	166	13.25%	138	13.80%	4	14.81%	24	10.62%	0.90

**Figure 1:**
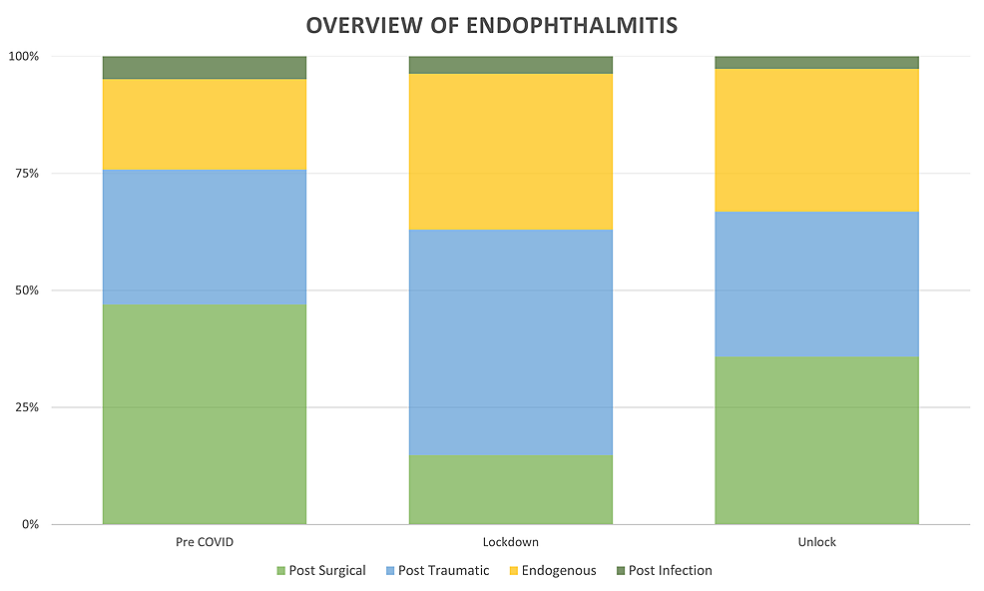
Overview of patients with endophthalmitis presenting during pre-COVID-19, lockdown (phases 1-4), and unlock (phases 1-10) in India

**Figure 2:**
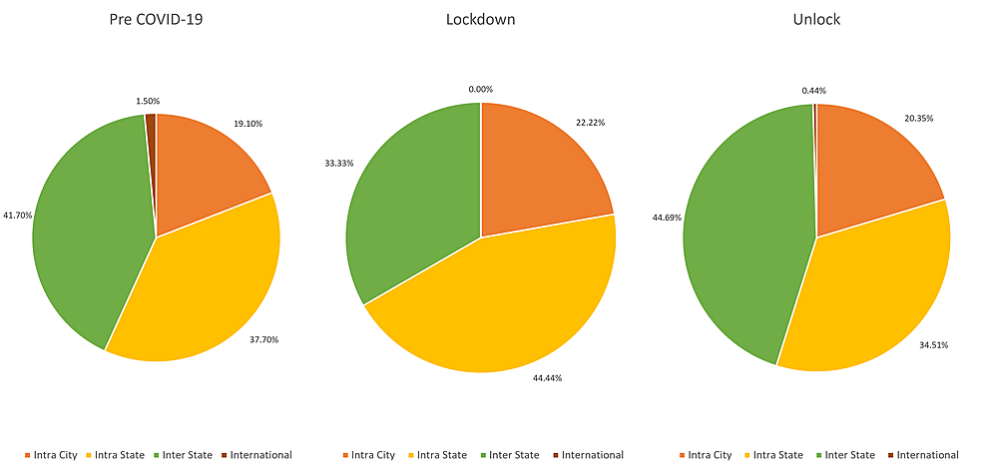
Geographic distribution of patients with endophthalmitis presenting during pre-COVID-19, lockdown (phases 1-4), and unlock (phases 1-10) in India

Compared to the pre-COVID-19 phase, with an average of 0.91 (1,000/1,096) patients per day, the number of patients seen during the lockdown phase with this diagnosis was significantly lower, with an average of 0.39 (27/68) per day, which increased to an average of 0.74 (226/303) during the unlock phase. There was a slight decrease in the mean age of the patients (42.38±22.86 years vs 43.74±23.72 years) and median (47 (IQR:25-61) years vs 51 (IQR:24-62) years) during the COVID-19 phase (lockdown and unlock phases) as compared to the pre-COVID-19 phase, which was statistically significant (p=<0.00001). There was a decrease (18.97%) in the proportion of pediatric patients (≤16 years) during the COVID-19 phase as compared to the pre-COVID phase (20.2%), which was not statistically significant (p=0.72). There was an increase in access to care among the male patients (70.75% vs 61.3%; p=0.19) and a decrease in female patients (29.25% vs 38.7%; p=0.05). There was a slight decrease in access to care among first-time visit (73.12% vs 74.5%; p=0.86) patients and an increase in follow-up patients (26.88% vs 25.5%; p=0.73), which was not statistically significant. There was a slight increase in patients with acute endophthalmitis (88.93% vs 86.2%; p=0.76) and a decrease in patients with chronic endophthalmitis (11.07% vs 13.8%; p=0.31), which was not statistically significant. The trend of patients with endophthalmitis over the three phases is detailed in Figure 1. With regards to the place of origin, a proportional reduction of 20.6% was seen in patients requiring inter-state travel and an increase was seen of 16.35% in intra-city and 18% in intra-state patients during the lockdown phase. There was a complete recovery in the proportion of outpatients to 107.17% requiring inter-state travel and incomplete recovery to 29.5% for international patients during the unlock phases. The detailed comparison of the geographic presentation in all three phases is described in Figure 2.

Specific trends of endophthalmitis

**Figure 3:**
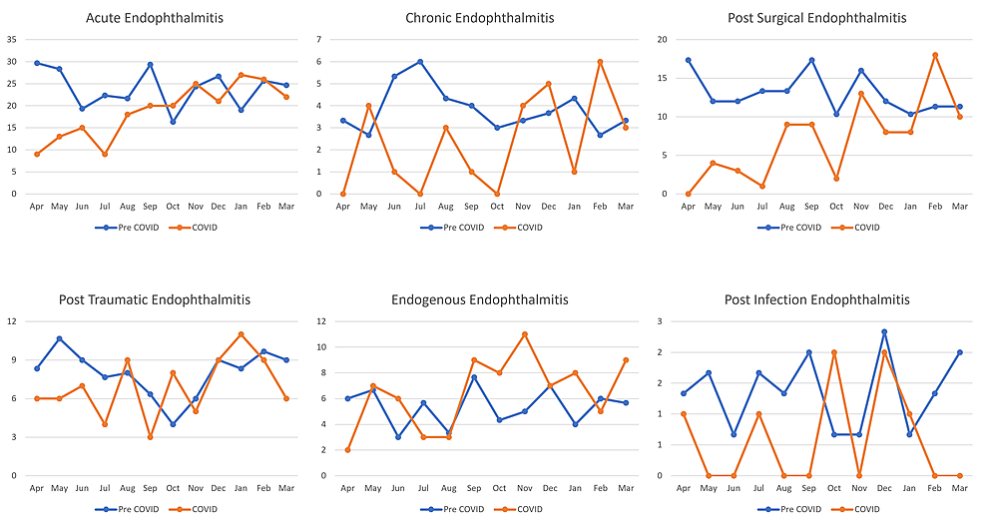
Monthly trends of patients with endophthalmitis during the pre-COVID-19 and COVID-10 phases in India

The averaged-annual frequency of endophthalmitis during the pre-COVID-19 phase was 333, which reduced to 253 during the COVID-19 phase, while the averaged-monthly frequency also reduced from 28 to 21 patients. For acute endophthalmitis, the averaged-annual frequency decreased from 287 to 225 patients, while the averaged-monthly frequency decreased from 24 to 19 patients. In chronic endophthalmitis, the averaged-annual frequency decreased from 46 to 28 patients, and from four to two patients for the averaged-monthly frequency. For post-surgical endophthalmitis, the averaged-annual frequency decreased from 157 to 85 patients, while the averaged-monthly frequency reduced from 13 to seven patients which exceeded the pre-COVID-19 monthly average by February 2021. The averaged-annual frequency of patients with post-traumatic endophthalmitis decreased from 96 to 83 patients, whereas the averaged-monthly frequency reduced from eight to seven patients and showed a gradually increasing trend to exceed the pre-COVID-19 monthly average by August 2020. The averaged-annual frequency of patients with endogenous endophthalmitis increased from 64 to 78 patients, whereas the averaged-monthly frequency increased from five to seven patients and exceeded the pre-COVID-19 monthly average by June 2021. For post-infection endophthalmitis, the averaged-annual frequency decreased from 16 to seven patients, while the averaged-monthly frequency reduced from two to one patient and never matched the pre-COVID-19 monthly average. A detailed comparison of the monthly trends of endophthalmitis in the pre-COVID-19 and COVID-19 phases is described in Figure 3.

## Discussion

This study sought to describe the impact of the lockdown and unlock phases of the COVID-19 pandemic on the presentation of patients with endophthalmitis at a tertiary eye care center in India. The study analyzed the pattern, distribution, and clinical profile of patients with endophthalmitis in the year one of the pandemic with the averages of the preceding three years to have a better comparative understanding. We observed that the first year of the COVID-19 pandemic saw a>50% decline in the number of patients with endophthalmitis accessing eye care services. There was an increase in the incidence of endogenous endophthalmitis and a decrease in post-trauma endophthalmitis. The unlock phase saw an increase in the patients from other states who were previously not able to access services.

Starting March 2020, most of the ophthalmologists in India were in a state of complete lockdown. Nair AG et al. published the results of a survey of the effect of COVID-19 on ophthalmic practices [10]. They noted that there was a near-total cessation of elective surgeries. Emergency services were being continued by 27.5% of ophthalmologists as per the survey. A significant number of ophthalmologists had switched over to teleconsultations to reach out to their patients. This though was a survey of the COVID-19 lockdown phase stand-alone and was not structured to gather comparative information regarding the status of the services before and after the lockdown. As this was a service overview type of survey, there was no detailed information about endophthalmitis in particular.

Posarelli C et al. conducted a study wherein they compared the charts of all patients that came to their emergency department during the lockdown period (March 10-May 4, 2020) with those in the same period of 2019 and the period from January 15-March 9, 2020 (pre-lockdown) [11]. They concluded that a significant reduction in visits during the lockdown was observed compared with those in the pre-lockdown period (reduction of 65.4%) and with those of the same period of 2019 (reduction of 74.3%). In their study, endophthalmitis accounted for 2% of all footfalls during the lockdown in the emergency clinic. The mean age of patients during the lockdown period was 55.5 ± 18.3 years and did not significantly change in the pre-lockdown period of 2020 (53.1 ± 19.4 years, P = 0.083) and with the March 10-May 4 period of 2019 (53.7 ± 21.1 years, P = 0.226) in their study. In the current study, in relation to endophthalmitis, there was a slight decrease in the mean age of the patients during the COVID-19 phase (lockdown and unlock phases) as compared to the pre-COVID-19 phase, which was statistically significant (p=<0.00001). Also, there was a decrease (18.97%) in the proportion of pediatric patients (≤16 years) during the COVID-19 phase as compared to the pre-COVID phase (20.2%), but this difference was not statistically significant.

Agarwal R et al. conducted a study to evaluate the impact of the COVID-19 pandemic, national lockdown, and unlocking on ophthalmic care provided by the government-funded apex health institute of India [12]. In their study, data regarding patients presenting to the ophthalmology department from March 23, 2020, to July 15, 2020, were compared with that from March 23, 2019, to July 15, 2019. The data between March 23, 2020, to May 31, 2020, (lockdown) and June 06, 2020, to July 15, 2020 (unlock) were also compared. They noted a decrease in the post-surgical endophthalmitis rates during the lockdown, from 2.38% to 1.46% of total patient footfall. This was attributed to the decrease in the overall surgeries performed during the lockdown. Their study did not elaborate on the differences in the etiology of endophthalmitis during the various periods of study.

In the current study, post-surgical endophthalmitis and post-trauma endophthalmitis reduced during the lockdown period as compared to the pre-lockdown. This could attribute to the decrease in the number of surgeries being performed during the lockdown phase. Shah S et al. described their results of an altered pattern of ocular trauma during the COVID-19 lockdown period [13]. In their study, the number of cases of ocular trauma requiring surgeries was 62/3106 (25.5%) in the pre-lockdown period, which changed to 52/483 (46.4%) in the lockdown phase. There, though the absolute number of trauma surgeries reduced during the lockdown, the proportion increased. This could be attributed to the relative decrease in elective cases. Their study did not elaborate specifically on post-trauma endophthalmitis.

The current study also demonstrated the month-wise trends of endophthalmitis patient footfalls in the pre-COVID phase in comparison to the lockdown and unlock phases. All etiologies of endophthalmitis showed a gradual increase in trends during the unlock phase. This matched the footfall in the pre-lockdown phase. The endogenous endophthalmitis subset showed a greater rebound of footfall, which was higher than that seen in the pre-COVID phase. This is attributable to the fact that a few of the patients following COVID-19 hospitalization developed endogenous endophthalmitis. Many centers in the unlock period have noted an increased incidence of endogenous endophthalmitis [14-15]. Prolonged steroid usage and intensive care unit course during COVID-19 treatment could explain the underlying reason for the rise of endogenous endophthalmitis. The unlock period also saw a gradual rise in the number of cases of post-trauma endophthalmitis. Most cases of post-trauma endophthalmitis occur due to penetrating trauma at the workplace or due to a violent incident outdoors [16-17]. As the lockdown period caused a natural reduction of such factors, it accounted for a reduction of these cases. Subsequent restoration of these demographic risk factors in the unlock period gave rise to an increase in the incidence of post-trauma endophthalmitis again.

The current study has a few limitations. As this study was a survey of records, it was retrospective in nature. Thus it would not have the power of a prospective observational study. The trends do not reflect the general trend of patients in the institute, as during COVID-19, only those patients who would feel an urgent need to visit the facility would have come, thus creating a sort of selection bias. Thus, this cannot be extrapolated to trends of footfall in an otherwise non-pandemic era.

## Conclusions

In conclusion, the first year of the COVID-19 pandemic saw a reduction to over half the number of patients seeking care for endophthalmitis during the lockdown phase. There was a significant increase in post-traumatic and endogenous endophthalmitis and a decrease in post-surgical cases during the lockdown phase. Many patients that required interstate travel to reach our facility could not reach us during COVID-19. Subsequently, their numbers recovered during the unlock phase. Prolonged usage of systemic steroids and other intravenous medications in COVID-19 patients resulted in an increased incidence of endogenous endophthalmitis, especially in the unlock period. As a lot of the world population remains to be vaccinated fully, this study gives a possible insight into the type of patients of endophthalmitis that a center can expect in an eventuality of a future wave of COVID-19 infection. This would hold the facility in good stead with respect to preparations needed to handle specific etiologies of endophthalmitis depending on the time frame of the pandemic.
